# Modeling the Effects of Morphine on Simian Immunodeficiency Virus Dynamics

**DOI:** 10.1371/journal.pcbi.1005127

**Published:** 2016-09-26

**Authors:** Naveen K. Vaidya, Ruy M. Ribeiro, Alan S. Perelson, Anil Kumar

**Affiliations:** 1 Department of Mathematics and Statistics, University of Missouri-Kansas City, Missouri, United States of America; 2 Division of Pharmacology, School of Pharmacy, University of Missouri-Kansas City, Missouri, United States of America; 3 Theoretical Biology and Biophysics Group, Los Alamos National Laboratory, Los Alamos, New Mexico, United States of America; Imperial College London, UNITED KINGDOM

## Abstract

Complications of HIV-1 infection in individuals who utilize drugs of abuse is a significant problem, because these drugs have been associated with higher virus replication and accelerated disease progression as well as severe neuropathogenesis. To gain further insight it is important to quantify the effects of drugs of abuse on HIV-1 infection dynamics. Here, we develop a mathematical model that incorporates experimentally observed effects of morphine on inducing HIV-1 co-receptor expression. For comparison we also considered viral dynamic models with cytolytic or noncytolytic effector cell responses. Based on the small sample size Akaike information criterion, these models were inferior to the new model based on changes in co-receptor expression. The model with morphine affecting co-receptor expression agrees well with the experimental data from simian immunodeficiency virus infections in morphine-addicted macaques. Our results show that morphine promotes a target cell subpopulation switch from a lower level of susceptibility to a state that is about 2-orders of magnitude higher in susceptibility to SIV infection. As a result, the proportion of target cells with higher susceptibility remains extremely high in morphine conditioning. Such a morphine-induced population switch not only has adverse effects on the replication rate, but also results in a higher steady state viral load and larger CD4 count drops. Moreover, morphine conditioning may pose extra obstacles to controlling viral load during antiretroviral therapy, such as pre-exposure prophylaxis and post infection treatments. This study provides, for the first time, a viral dynamics model, viral dynamics parameters, and related analytical and simulation results for SIV dynamics under drugs of abuse.

## Author Summary

The HIV epidemic continues to be a severe public health problem within the USA and many parts of the world. A substantial number of HIV-infected individuals are intravenous drug users who abuse opiates. Drugs of abuse lead not only to high HIV transmission, but also to high viral load, increased disease progression, and augmented HIV-associated neurocognitive disorder in HIV-1 infected individuals. In this study, using a novel mathematical model and experimental data from macaques, we evaluate some important effects of morphine, an active metabolite of heroin, on simian immunodeficiency virus (SIV) dynamics. In particular, we quantify the morphine-induced target cell subpopulation switch that results in a high proportion of target cells with higher susceptibility to SIV infection. Consistent with experimental observations, our results reveal that morphine promotes increased co-receptor expression, which can increase the rate of target cell infection. This in turn may explain the observation that HIV infected individuals with drugs of abuse may suffer from higher viral load, higher CD4 count loss, a larger basic reproduction number, and higher probability of failing antiretroviral therapy. Our model and results may be useful to formulate guidelines on incorporating extra efforts needed to manage HIV infection in drug abusers.

## Introduction

Drug abuse is one of the leading causes for transmission of human immunodeficiency virus (HIV) within the USA, and in many parts of the world [1–3]. Since the beginning of the HIV epidemic, injection drug use (IDU) has contributed more than one-third (36%) to the total AIDS cases in the United States [4]. HIV-1 infected patients often suffer from HIV-associated neurocognitive disorder (HAND), which has been substantially linked with drug abuse [3, 5]. Contaminated needle sharing and increased risky sexual behavior among injection drug users put them at a higher risk of getting infected with HIV and at a greater chance of developing neurological abnormalities [6–8]. The dependence of many HIV-infected patients on drugs of abuse, such as opiates, is a significant problem in the post-HAART era [3]. Despite being a considerable problem for both science and society, the mechanism by which drug abuse contributes to HIV infection and pathogenesis is not well understood and it is important to study how drugs of abuse affect HIV dynamics.

Opiate addictions often include many uncertain factors such as variable poly-drug abuse, inconsistent drug dosing, poor subject health, risky sexual behavior, variable stressful environment, and the use of other illicit substances [9]. As a result of such extreme complexity of the opiate addiction, studies on HIV infection combined with opiate addictions have reached contrasting conclusions, including no effects, adverse effects, and survival advantages for those engaged in opiate abuse [10–14].

Better understanding of the effects of drugs of abuse, such as morphine, on viral pathogenesis and disease progression has been provided by the development of experimental nonhuman primate models of IDU and HIV disease that utilize simian immunodeficiency virus (SIV) and simian human immunodeficiency virus (SHIV) in rhesus macaques [3, 15–17]. The experiments utilizing these animal models along with other in-vitro experiments demonstrate considerable effects of morphine on viral dynamics, immune responses, and virus evolution [3, 15–20]. Combined these experiments strongly support the hypothesis that morphine enhances HIV-1/SIV infectivity as well as HAND.

Mathematical models have significantly contributed to our understanding of virus and immune cell dynamics [21–24], and may become valuable in understanding HIV infection dynamics under drugs of abuse. Here, we develop a novel viral dynamic model with the main objective of studying whether a model based on the experimental observation that morphine upregulates the expression of SIV/HIV co-receptors [25–27] can explain the effect of morphine on SIV viral load kinetics in morphine-dependent SIV-infected macaques. We show that our model agrees well with viral load and CD4+ T-cell count data from both morphine-dependent and control macaques infected intravenously with a mixture of SHIV and SIV. Using our model, we evaluate the role of morphine in altering target cell susceptibility, viral dynamics, steady-state viral load, the loss of CD4 cells, and the effectiveness of antiretroviral therapy (ART). We further discuss how morphine can increase the basic reproduction number, causing further obstacles for HIV prophylaxis.

## Materials and Methods

### Ethics Statement

Rhesus macaques used for the study were obtained from the Caribbean Research Primate Center and housed in the Animal Resource Center of the University of Puerto Rico, San Juan. The experimental protocol was approved by the Institutional Animal Care and Use Committee, and the research was performed in accordance with the Guide for the Care and Use of Laboratory Animals.

### Experiment and Data

Viral load and CD4 count data were obtained from 12 male rhesus macaques (*Macaca mulatta*) [16]. The animals were confirmed negative for simian T-cell leukemia virus type 1 and simian retrovirus. The animals were divided into two groups: morphine-dependent (6 animals) and control (6 animals). The morphine-dependent environment was created and maintained by injecting intramuscularly three daily 1–5 mg/kg doses of morphine for 2 weeks, followed by three daily 5 mg/kg doses for an additional 18 weeks. The same amount of normal saline at the same time was given to control animals. All macaques were infected intravenously with a 2-ml inoculum containing 10^4^ TCID_50_ doses each of SHIV_KU-1B_, SHIV_89.6p_, and SIV 17E-Fr. These animals were monitored for a period of 12 weeks, and the plasma viral load and CD4 count were measured at weeks 0, 1, 2, 3, 4, 6, 8, 10, and 12 post infection as described in Kumar et al. [16]. The morphine- dependent animals were maintained on morphine throughout the observation period. The experimental data are given in Table 1.

**Table 1:** Viral loads (vRNA copies/ml) and CD4 counts (CD4 cells per *μl*) for individual monkeys (Morphine group: M1-M6; Control group: C1-C6).

Animal		Wk 0	Wk 1	Wk 2	Wk 3	Wk 4	Wk 6	Wk 8	Wk 10	Wk 12
M1	VL		6.67e5	4.93e6	4.79e4	1.20e5	4.79e4	3.47e5	4.85e6	4.01e6
	CD4	852	133	45	24	45	57	6	10	16
M2	VL		7.83e5	3.57e7	8.63e5	2.03e5	3.55e5	4.95e5	1.65e6	2.03e7
	CD4	1373	617	185	56	36	9	9	21	6
M3	VL		1.52e7	1.81e7	5.10e5	6.40e5	5.10e5	1.60e7	7.32e6	2.59e7
	CD4	1193	164	34	17	22	128	4	7	39
M4	VL		3.55e6	2.41e6	3.08e5	1.36e5	2.10e4	2.3e3	5.6e3	1.4e3
	CD4	1263	77	422	243	274	552	676	687	707
M5	VL		1.67e7	7.46e6	2.95e6	3.12e5	4.64e5	2.46e5	1.92e5	4.06e5
	CD4	1316	43	65	112	440	29	25	31	42
M6	VL		5.29e6		2.12e5	2.80e5	5.26e5	5.08e4	1.89e5	1.28e5
	CD4	1281	191	16	5	5	137	4	8	31
C1	VL		7.57e6	8.96e6	5.89e5	8.32e5	2.75e5	9.84e4	2.25e5	3.14e4
	CD4	1567	609	399	175	536	469	448	656	334
C2	VL		2.03e7	2.79e6	9.20e4	9.70e4	1.13e5	7.50e4	9.5e4	8.70e4
	CD4	1779	453	247	49	70	150	191	369	113
C3	VL		1.29e6	1.55e7	3.29e6	3.37e5	1.86e5	9.10e4	4.8e4	1.4e4
	CD4	751	340	559	55	110	98	102	135	154
C4	VL		1.38e7	1.44e7	8.32e5	7.32e5	6.49e5	4.22e5	4.22e5	2.56e5
	CD4	671	665	315	380	410	380	445	448	510
C5	VL		4.04e6	2.03e7	3.19e5	1.50e5	8.00e4	5.50e4	3.30e4	7.00e3
	CD4	1642	765	400	169	188	231	263	294	264
C6	VL		4.06e6	5.66e6	3.77e5	1.42e5	3.34e5	2.03e5	1.07e5	1.75e5
	CD4	1414	885	307	82	101	119	171	373	102

### Model

We incorporate experimentally observed morphine effects into the basic model of viral infection that has been used previously to describe acute infection in both HIV infected humans and SIV infected macaques [28–33]. We focus on modeling acute infection dynamics during the first 84 days post infection, when viral load and CD4+ T cell counts were obtained from the experimentally infected macaques. During the early period, neutralizing antibodies specific to any of the SIV strains were not detected (Kumar’s Lab). Further, during this period, no significant difference in antibody and CD8+ T-cell levels were found between the morphine-dependent and control animal groups (Kumar’s Lab). Thus, while immune effects may occur during this early period [34], and some viral kinetic models of acute infection have included immune responses [35–40], the available data suggests that they are not responsible for the difference in viral dynamics between the morphine-dependent and control group. For this reason, as well as the fact that target cell limited models have been shown to agree well with viral load data collected during acute infection, we choose as our base model a target cell limited model, as done in previous studies of acute infection [28–32]. However, we also examined alternative explanations based on an effect of morphine on the immune response.

The direct correlation between morphine dependence and viral replication has been attributed to the increased expression of chemokine receptors, such as CCR5 and CXCR4, on lymphocytes and other cells [3, 26, 27, 41]. To successfully infect target cells, HIV requires co-receptors, mainly CCR5 or CXCR4, along with CD4 receptors [42, 43]. HIV infectivity, as measured by HIV p24 levels, is proportional to co-receptor expression [44]. Experimental studies have clearly demonstrated that morphine increases CCR5 and CXCR4 expression on various cell types [26, 27, 41, 45, 46], and can lead to higher set-point viral loads [16]. Combined, these experimental results suggest that morphine by inducing HIV co-receptor expression on target cells increases the susceptibility of these cells to HIV infection, resulting in higher viral replication. We note that there has been limited study of co-receptor expression *in vivo* under morphine conditioning. Thus the objectives of this study are to model the hypothesis that morphine increases CCR5 expression, to test this hypothesis in a quantitative manner and to see if it yields a model that fits *in vivo* data.

Based on *in vitro* observations about the effects of morphine conditioning on CCR5 levels, we develop a model containing two subpopulations of target cells (CD4^+^ T cells)—one with lower susceptibility to infection (i.e. lower infection rate) due to a low level of co-receptor expression, *T*_*l*_, and another with higher susceptibility (i.e. higher infection rate) due to a high level of co-receptor expression, *T*_*h*_. Levels of co-receptor expression could more accurately be described by considering many subpopulations of target cells, but the data we analyze lacks such precision, so we opted for this simpler approach. Further, as cells become activated and differentiate, co-receptor expression can change. Thus, our model incorporates a base rate at which cells transfer from low expression to high expression. A key hypothesis to be tested is that morphine enhances the rate of cells transferring from the *T*_*l*_-group to the *T*_*h*_-group resulting in a higher proportion of *T*_*h*_ cells in the target cell population.

**Fig 1:**
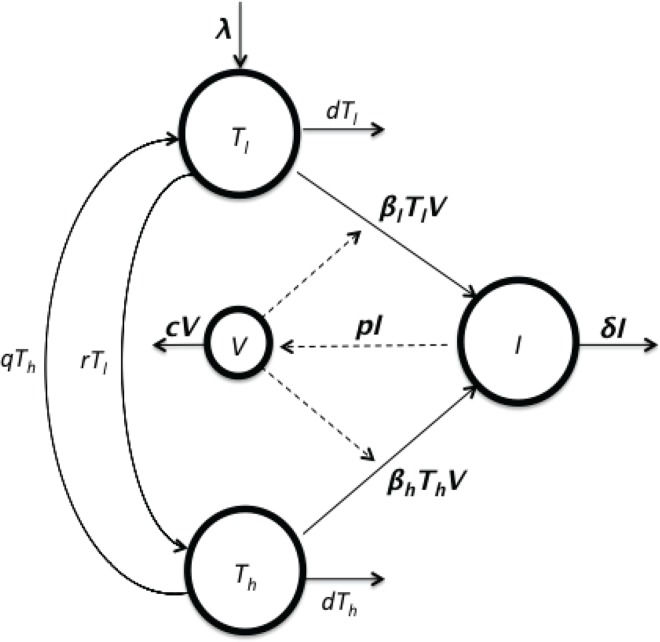
Schematic diagram of the model. The model contains two subpopulations, *T*_*l*_ and *T*_*h*_ of target cells, with low and high susceptibilities to infection. Cells within these populations can switch susceptibilities with rates *r* and *q*, respectively. The target cells are infected, upon contact with virus, *V*, at rates *β*_*l*_ and *β*_*h*_, respectively, and become productively infected cells, *I*.

A schematic diagram of the model is presented in Fig 1. We assume that target cells are generated at a constant rate *λ* and have a per capita net loss rate *d*, which is the difference between the rate of loss from cell death and rate of gain due to cell division. For simplicity, we assume that newly generated target cells are all in the *T*_*l*_-group. The rate of transition from *T*_*l*_ to *T*_*h*_ is denoted by *r*, while that from *T*_*h*_ to *T*_*l*_ is denoted by *q*. Target cells, *T*_*l*_ and *T*_*h*_, become productively infected cells, *I*, upon contact with free virus, *V*, at rates *β*_*l*_ and *β*_*h*_, respectively. The parameters *δ*, *p*, and *c* are the rate constants of infected cell loss, virus production by infected cells, and virus clearance, respectively. The model can be described by the following set of equations:

$$\frac{dT_{l}}{dt}=\lambda +qT_{h}-dT_{l}-rT_{l}-\beta_{l}VT_{l},\;T_{l}(0)=T_{l0},$$ (1)

$$\frac{dT_{h}}{dt}=rT_{l}-dT_{h}-\beta_{h}VT_{h}-qT_{h},\;T_{h}(0)=T_{h0},$$ (2)

$$\frac{dI}{dt}=\beta_{l}VT_{l}+\beta_{h}VT_{h}-\delta I,\;I(0)=I_{0},$$ (3)

$$\frac{dV}{dt}=pI-cV,\;V(0)=V_{0}.$$ (4)

In this model, the total CD4 count is given by *T* = *T*_*l*_ + *T*_*h*_ + *I*, with *T(0) = T*_*0*_. The change in CD4 count is thus governed by the equation *dT*/*dt* = *λ* − *dT* − (*δ* − *d*)*I*.

As mentioned above, we also examined immune response models (IR). The details of these models are given in S2 Text. In summary, we modified the basic viral dynamics model ($\frac{dT}{dt}=\lambda -dT-\beta VT,\;\frac{dI}{dt}=\beta VT-\delta I,\;\frac{dV}{dt}=pI-cV$), by introducing an equation, $\frac{dE}{dt}=\frac{\xi I}{K+I}E-d_{E}E$, representing the dynamics of effector cells, *E*. We then considered two different effects of the immune response on the viral dynamics, namely cytolytic (CIR model) and non-cytolytic (NIR model) effects. We modeled the cytolytic effect by replacing *δ* by (*δ* + *δ*_*E*_*E*), and the non-cytolytic effect by replacing *p* by *p*/(1 + *p*_*E*_*E*), where *δ*_*E*_ and *p*_*E*_ represent the rate of killing of infected cells (cytolytic) and the rate of reduction of viral production (non-cytolytic) due to cytokines released by effector cells, respectively. We fitted these models to each group of animals separately, as well as to both groups combined, and compared the models using the small-sample (second order) Akaike information criterion [47], *AIC*_*C*_ (see S2 Text). The smallest *AIC*_*C*_ value obtained among all cases of the CIR and NIR models was 14.97, while the *AIC*_*C*_ value for our target cell population switch (TCS) model is -5.07 (morphine) and -15.59 (control). Moreover, the killing rate *δ*_*E*_*E* predicted by the CIR model is always smaller than *δ*, and the factor *p*_*E*_*E* predicted by NIR model is much smaller than 1, indicating that the putative effects of the immune response effects are negligible. In S2 Text we discuss these immune response models, show the best fit parameters and present the fits to the data.

### Parameter Estimation

Since the animals were initially uninfected, we set *I*_0_ = 0. As estimated by Ramratnam et al. [48], the virion clearance rate constant during chronic infection in humans varies between 9.1 and 36.0 day^-1^, with an average of 23 day^-1^. Results in Zhang et al. [49] indicate that SIV clearance rate from plasma of rhesus macaques is at least as fast. Here, we take *c* = 23 day^-1^ as a minimal estimate and acknowledge that this value might be larger in macaques. Following previous estimates [32], we take 100 days as the life span of uninfected target cells, i.e., *d* = 0.01 day^-1^. Initially, the average CD4 counts in the control and morphine-conditioned animals were 1,366 per *μl* and 1,213 per *μl*, respectively. According to the vitro experiment by Steele et al. [41], about 3% of the control cells and about 5% of the morphine treated cells expressed CCR5 in the absence of infection. This allows us to estimate that there are 40,980 and 60,650 CCR5 expressing CD4+ T cells/ml in the absence of infection in this in vitro system. Due to lack of in vivo co-receptor expression data, we take these values as initial estimate of cells with higher CCR5 expression. That is, we take *T*_*h*0_ = 40,980/*ml*, *T*_*l*0_ = *T*(0) − *T*_*h*0_ for the control group, and *T*_*h*0_ = 60,650/*ml*, *T*_*l*0_ = *T*(0) − *T*_*h*0_ for the morphine group. We also performed a sensitivity analysis on the effect of changing *T*_*h*0_.

Note that as shown by Sachsenberg et al. [50], only a fraction of CD4^+^ T cells in peripheral blood express activation markers and hence are preferred targets for HIV infection. Based on this, Stafford et al. [32] chose to use 1% of all CD4^+^ T cells as targets for HIV. However, the fraction of CD4^+^ T cells chosen to be the initial number of target cells, *T*_0_, does not affect the results of this study because the only parameters affected by a different choice of *T*_0_ are *λ* and *p*, and the redefining *T*_*l*_ → *T*_*l*_/*T*_0_, *T*_*h*_ → *T*_*h*_/*T*_0_, *I* → *I*/*T*_0_, *λ* → *λT*_0_, and *p* → *p*/*T*_0_ will keep the system unaltered (see S1 Text). The scaled system shows that the estimate of *p* is related to *T*_0_ as *pT*_0_ appears in the scaled system instead of *p*. Using the SIV burst size *in vivo* in rhesus macaques as approximately 5 × 10^4^ virions per infected cell [51], an average life-span of productively infected cells of 1 day [52], and the previous estimate of the number of target cells for SIV infection in macaques of 5% of the CD4 count [33], we take *p* = 2500 d^-1^.

The initial viral load, *V*_0_, needs to be estimated. Each macaque was infected intravenously with a 2-ml inoculum containing 10^4^ TCID_50_ of each of three chosen SIV viruses [16]. The total of 3 × 10^4^ TCID_50_ of viruses comprises at least 3 × 10^5^ HIV RNA copies [53]. A macaque, on average, weighs 1/10 of a human, which approximately gives 1.5 liter of extracellular water in a macaque. Assuming that the infused virions (RNA copies) are dispersed into extracellular water, the initial viral load, *V*_0_, can be estimated as *V*_0_ ≈ 3 × 10^5^/1.5*L* ≈ 200 viral RNA copies/ml. Thus we take *V*_0_ = 200 viral RNA copies/ml for the base case and perform a sensitivity analysis by varying *V*_0_ from 1 log_10_ to 4 log_10_ viral RNA copies/ml. We estimated the remaining parameters (*λ*, *β*_*l*_, *β*_*h*_, δ, *r*, *q*) by fitting the model to experimental data.

### Data Fitting

We performed data fitting to each monkey individually. We solved Eqs (1–4) numerically using the Runge-Kutta 4 algorithm in Berkeley Madonna [54]. The predicted log_10_ values of the viral loads and the CD4 counts were fit to the corresponding log-transformed data via a nonlinear least squares regression method, in which the sum of the squared residuals (*SSR*), i.e. the difference between the model predictions and the corresponding experimental values, is minimized. *SSR* is calculated using the following formula:

$$SSR=\sum_{t_{V}=1}^{N_{V}}{\left[\log_{10}\;V(t_{V})-\log_{10}\;\bar{V}(t_{V})\right]}^{2}+\sum_{t_{C}=1}^{N_{C}}{\left[\log_{10}\;T(t_{C})-\log_{10}\;\bar{T}(t_{C})\right]}^{2},$$

where *V*(*t*_*V*_) represents the viral load at time *t*_*V*_ predicted by the model, *T*(*t*_*C*_) represents CD4 count at time *t*_*C*_ predicted by the model, and $\bar{V}(t_{V})$ and $\bar{T}(t_{C})$ are the corresponding data. *N*_*V*_ and *N*_*C*_ are total number of viral load and CD4 count data points used for fitting, respectively. In our case, a total of 15 to 16 data points are available for each fitting. As the maximum viral load and maximum T cell count per mL were similar we did not weight the two terms on the right-hand side of this equation differently.

We also tried fitting viral load on a log_10_ scale and CD4 count on a linear scale, but we found that the best-fit parameters were highly dependent on the ratio of weights, applied to the sum of residuals of these two data sets.

Using the set of parameters obtained from Berkeley Madonna as initial guesses, we refined the fit using MATLAB. Finally, for each best-fit parameter estimate, we provide 95% confidence intervals (CI), which were computed from 1000 replicates, by bootstrapping the residuals [55, 56]. We used t-tests to compare the estimated parameters for the two groups of animals.

## Results

Based on experimental studies that showed increased levels of CCR5 and CXCR4 expression in various cell types [26, 27, 41, 45, 46] in morphine-dependent animals and a high correlation between co-receptors expression and the pathogenesis of HIV and SIV infection [41, 57–59], we developed a model to test the hypothesis that morphine increases the susceptibility of target cells to HIV/SIV infection. The model contains two subpopulations of target cells (CD4^+^ T cells) with lower and higher susceptibilities to infection due to different level of co-receptor expression.

### Model-Fit to the Data

**Fig 2:**
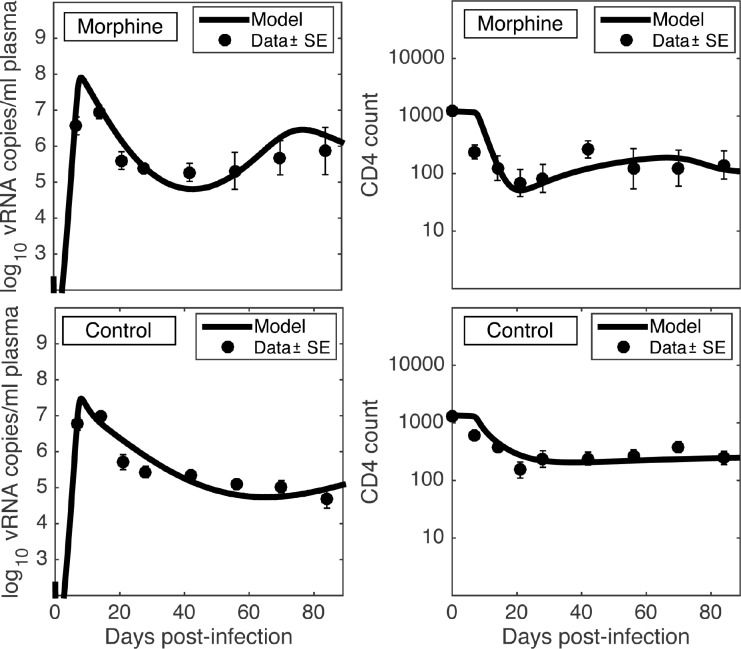
Model fit to the data. Best-fit viral load (left column) and CD4 count/microliter (right column) dynamics predicted by the model (solid line) along with the mean *log*_10_ viral load and the mean CD4 count data (filled small circles with bars representing standard errors) for the morphine group (first row) and the control group (second row). Parameters used, including the best estimates for model parameters, are given in Table 2.

Estimated parameters obtained by fitting the data from the individual macaques in the morphine group and the control group are given in Table 2 and the corresponding best-fits to the data in each animal are shown in S1 Fig. 95% bootstrap confidence intervals of the estimated parameters are given in S1 Table. In order to make a visual comparison between the viral load kinetics in the two groups of animals more apparent, we plot in Fig 2 the mean *log*_10_ viral load and mean CD4 count dynamics for the two groups as well as best-fit curves to the mean data. Consistent with previous experimental results [16], as shown in Fig 2, the model predicts a significantly higher viral load set-point in the morphine group than in the control group. Moreover, morphine dependence may alter various properties related to SIV dynamics, which we analyze in the subsections below, including the variation of these properties among the animals.

**Table 2:** Estimated Parameters for Individual Monkeys (Morphine group: M1-M6; Control group: C1-C6). Last row for each group represents estimates for the mean *log*_10_ viral load and the mean CD4 count.

Monkey	*δ*	*λ*	*r*	*q*	*β*_*l*_	*β*_*h*_
	day^-1^	ml/day	day^-1^	day^-1^	ml/day	ml/day
M1	0.36	1.97 × 10^3^	0.62	4.91 × 10^−7^	6.15 × 10^−9^	7.63 × 10^−8^
M2	0.31	2.74 × 10^3^	0.56	4.97 × 10^−7^	4.83 × 10^−10^	9.60 × 10^−8^
M3	0.39	9.42 × 10^3^	0.39	4.83 × 10^−6^	5.83 × 10^−9^	7.88 × 10^−8^
M4	0.62	9.39 × 10^3^	0.70	2.40 × 10^−7^	9.79 × 10^−9^	1.79 × 10^−8^
M5	0.53	3.41 × 10^3^	0.30	4.53 × 10^−8^	1.52 × 10^−11^	9.81 × 10^−8^
M6	0.31	1.47 × 10^3^	0.53	4.64 × 10^−8^	5.22 × 10^−10^	9.80 × 10^−8^
Mean	0.42	4.73 × 10^3^	0.52	1.02 × 10^−6^	3.80 × 10^−9^	7.75 × 10^−8^
Median	0.37	3.08 × 10^3^	0.55	3.66 × 10^−7^	3.18 × 10^−9^	8.74 × 10^−8^
SD	0.12	3.68 × 10^3^	0.15	1.87 × 10^−6^	4.04 × 10^−9^	3.08 × 10^−8^
Mean Data	0.38	5.18 × 10^3^	0.50	4.42 × 10^−7^	5.13 × 10^−10^	3.02 × 10^−8^
C1	0.77	3.69 × 10^3^	0.13	0.22	1.05 × 10^−11^	7.75 × 10^−8^
C2	0.77	3.63 × 10^3^	0.18	0.22	7.41 × 10^−10^	7.50 × 10^−8^
C3	0.41	1.92 × 10^3^	0.18	0.28	6.25 × 10^−10^	9.88 × 10^−8^
C4	0.68	4.27 × 10^3^	0.14	0.23	5.09 × 10^−10^	1.66 × 10^−7^
C5	0.78	4.11 × 10^3^	0.16	0.26	4.14 × 10^−11^	6.49 × 10^−8^
C6	0.74	3.47 × 10^3^	0.14	0.24	8.61 × 10^−11^	8.63 × 10^−8^
Mean	0.69	3.51 × 10^3^	0.16	0.24	3.36 × 10^−10^	9.48 × 10^−8^
Median	0.75	3.66 × 10^3^	0.15	0.23	2.98 × 10^−10^	8.19 × 10^−8^
SD	0.14	8.38 × 10^2^	0.02	0.02	3.26 × 10^−10^	3.67 × 10^−8^
Mean Data	0.78	3.69 × 10^3^	0.16	0.24	1.10 × 10^−10^	8.42 × 10^−8^
p-value	0.013	0.70	0.0049	0.005	0.24	0.94

### Susceptibility of T Cell Subpopulations

In our model we considered two classes of target cells, *T*_*l*_ and *T*_*h*_, with different susceptibilities to HIV/SIV infection due to different levels of co-receptor expression. We estimated the infection rates *β*_*l*_ and *β*_*h*_, corresponding to the infection of *T*_*l*_ and *T*_*h*_ cells, respectively (Table 2). We found that *β*_*h*_ is significantly higher than *β*_*l*_ (1 to 2 orders of magnitude higher in both the morphine and control groups), showing that *T*_*h*_ target cells are more susceptible to SIV infection than *T*_*l*_ target cells. This difference between *β*_*l*_ and *β*_*h*_ is statistically significant in the morphine group (t-test, p<0.001), the control group (t-test, p<0.001), as well as all animals combined (t-test, p<0.001). The infection rate increase estimated here is in agreement with an experiment in which an increase in cells expressing CCR5 from 1.75% to 12.5% increased the p24 level by 2 orders of magnitude [44]. Note that the differences in *β*_*l*_ between the two groups and *β*_*h*_ between the two groups are not statistically significant (t-test, p > 0.05 for both tests, Table 2). This shows that there is a minimal contribution of variation in susceptibility among animals to the difference in infection susceptibility between two groups of target cells.

### Effects of Morphine on Target Cell Subpopulation Switch

In our model, the rate of target cell subpopulation switch is represented by two parameters: *r*, the transfer rate from the *T*_*l*_ compartment to the *T*_*h*_ compartment, and *q*, the transfer rate from the *T*_*h*_ compartment to the *T*_*l*_ compartment. Our estimates indicate that morphine causes target cells to transfer from the *T*_*l*_ compartment to the *T*_*h*_ compartment about 3 times faster compared to the control environment [*r* = 0.52±0.02 per day in the morphine group vs. *r* = 0.16±0.01 per day in the control group]. The difference in *r* between two groups is statistically significant (t-test, p<0.005). More importantly, the backward transfer rate, *q*, is significantly lower (t-test, p = 0.005) in the morphine group compared to the control group [*q* = 1.02 ± 0.75 × 10^−6^ per day in the morphine group vs. *q* = 0.24±0.01 per day in the control group]. These parameter estimates strongly suggest that morphine can have an important role in altering the nature of target cells. The results are also in agreement with previous *in vitro* [27, 41] and *in vivo* [60] experiments that show morphine promotes co-receptor expression in target cells thereby increasing their susceptibility to HIV/SIV infection.

### Basic Reproduction Number

The basic reproduction number, denoted by *R*_0_, is an important measure of host viral dynamics, as it determines whether a virus can establish infection [21, 61, 62]. *R*_0_ is defined as the average number of cells infected by a single infected cell when there is no target cell limitation. In general, if *R*_0_ < 1 the infection will die out, and if *R*_0_ > 1 the infection will spread [21, 61, 62]. Using the next-generation method (see S1 Text), we derive the basic reproduction number of our model as:

$$R_{0}=\frac{\lambda p}{\delta cd(d+r+q)}[\beta_{l}(d+q)+\beta_{h}r]$$ (5)

Substituting our parameter estimates into Eq (5), we obtained *R*_0_ = 2.08 for the control group and *R*_0_ = 9.69 for the morphine group. With *R*_0_ > 1 in both groups, our model predicts that the infection spreads in both groups, consistent with the data. Moreover, these estimates show that the morphine can significantly affect the basic reproduction number.

### Long-Term Effects of Morphine

#### In the absence of SIV infection

In the absence of SIV infection, i.e. *V* = *I* = 0, the model reduces to

$$\frac{dT_{l}}{dt}=\lambda +qT_{h}-dT_{l}-rT_{l},\;T_{l}(0)=T_{l0},$$ (6)

$$\frac{dT_{h}}{dt}=rT_{l}-dT_{h}-qT_{h},\;T_{h}(0)=T_{h0},$$ (7)

Further, in the absence of infection, the total T cells in circulation remains approximately constant, i.e. $\frac{d(T_{l}+T_{h})}{dt}=0$, giving *λ* = *d*(*T*_*l*_ + *T*_*h*_). In this case, the proportion of *T*_*h*_ cells, *x* = *T*_*h*_/(*T*_*l*_ + *T*_*h*_), is given by the solution of

$$\frac{dx}{dt}=r-(d+r+q)x$$ (8)

Solving Eq (8), we obtain the time-dependent solution for the proportion of *T*_*h*_ cells as

$$x(t)=\frac{r}{\left(d+r+q\right)}+\left[x(0)-\frac{r}{\left(d+r+q\right)}\right]e^{-(d+r+q)t}$$ (9)

Taking the limit as *t* → ∞ in Eq (9), we obtain that in long-term morphine conditioning, the proportion of *T*_*h*_ cells reaches *r*/(*d* + *r* + *q*) in the absence of infection.

#### In the presence of SIV infection

To understand the effects of morphine on the long-term dynamics during SIV infection, we analyze two possible steady states, the infection-free steady state, *E*^0^, and the infected steady state, E*. The infection free steady state, is given by

$$E^{0}=(T_{l}^{0},T_{h}^{0},I^{0},V^{0})=\left(\frac{\lambda (d+q)}{d(d+r+q)},\frac{\lambda r}{d(d+r+q)},0,0\right).$$

Linearizing the system around *E*^0^, and analyzing the eigenvalues of the Jacobian matrix, we can prove that *E*^0^ is asymptotically stable if *R*_0_ < 1 and unstable if *R*_0_ > 1 (see S1 Text) as discussed above.

For the analysis of the infected steady state during morphine conditioning, we assume *β*_*l*_ ≪ *β*_*h*_ and *q* ≪ *r*, as given by our parameter estimates, in order to simplify the model. In this case, E* is given by

$$E^{*}=(T_{l}^{*},T_{h}^{*},I^{*},V^{*})=\left(\frac{\lambda }{d+r},\frac{\delta c}{\beta_{h}p},\frac{dc}{\beta_{h}p}(R_{0}-1),\frac{d}{\beta_{h}}(R_{0}-1)\right).$$

Clearly, E* exists if *R*_0_ > 1. Furthermore, we are able to prove that E*, if it exists, is asymptotically stable (see S1 Text). This expression shows that a higher value of *R*_0_ as in morphine group gives a higher equilibrium viral load.

**Fig 3:**
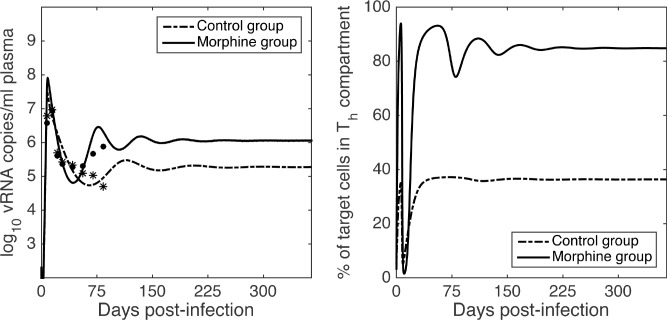
Long term dynamics of viral load and T cell subpopulations. Long term dynamics, predicted by the model, of the viral load (left) and the % of target cells that are *T*_*h*_ cells (right) for the morphine group (dashed-dot curve) and the control group (solid curve). Parameters given in Table 2 are used for model simulations. Small circles indicate the available data, which is restricted to time points early in infection.

We then carried out simulations of the full model to analyze effects of morphine on the dynamics as well as the infected-steady state level. For about 5–6 weeks post-infection, the plasma viral load dynamics remained generally comparable in both the morphine and control groups. However, after 6 weeks of infection the viral load in the morphine group increases and eventually approaches a higher steady state level (Fig 3). In contrast to the morphine group, the viral load in the control group did not increase and leveled off with about a 1.0 log_10_ lower steady state value. We also observed how the percentage of target cells in *T*_*h*_ compartment changes over time (Fig 3). In both groups, the percentage of target cells in the *T*_*h*_ compartment decreases after infection following a brief delay, reaches a minimum, and again increases to a steady state level. Throughout the dynamics, this percentage in the morphine group almost always remained higher than in the control group, with a significantly higher steady state level in the morphine group (Fig 3).

**Fig 4:**
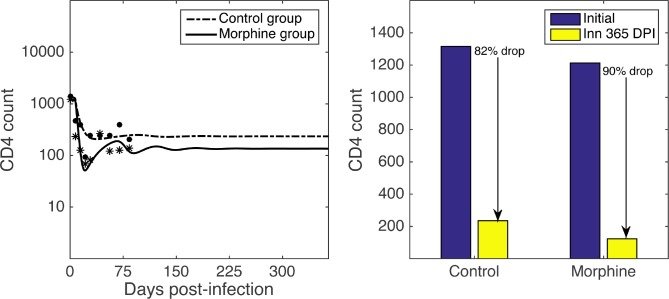
CD4 count dynamics. Model prediction for the long term dynamics of CD4 count (left) and the total loss of CD4 count during a year of SIV infection (right) for the morphine group (dashed-dot curve) and the control group (solid curve). Parameters given in Table 2 are used for model simulations. Small circles indicate the available data.

Since morphine conditioning results in a higher steady state level of target cells in the *T*_*h*_ compartment, which have a higher infection rate, morphine was expected to cause a greater loss of CD4 cells. To quantify this, we simulated the dynamics of CD4 cells (Fig 4) and computed the total loss of CD4 cells during one year of SIV infection. Our model predicts a rapid decrease of the CD4 count in the morphine group compared to the control group at the beginning of the infection, consistent with the experimental data. At the end of a year, the predicted total CD4 count drop is 90% in the morphine group while it is 82% in the control group (Fig 4). The difference in CD4 loss between the two groups is not statistically significant (t-test, p > 0.05). However, animal M4 has an extremely high CD4 count throughout the infection (see Table 1, S1 Fig); and its set point CD4 count remains higher than 700 cells/μL while the maximum set point CD4 count of all other animals in the morphine group is 42 cells/μL. Excluding animal M4, the loss of CD4 cells in the morphine group is significantly higher than that in the control group (t-test, p < 0.001).

### Effect of Morphine in the Presence of Antiretroviral Therapy

**Fig 5:**
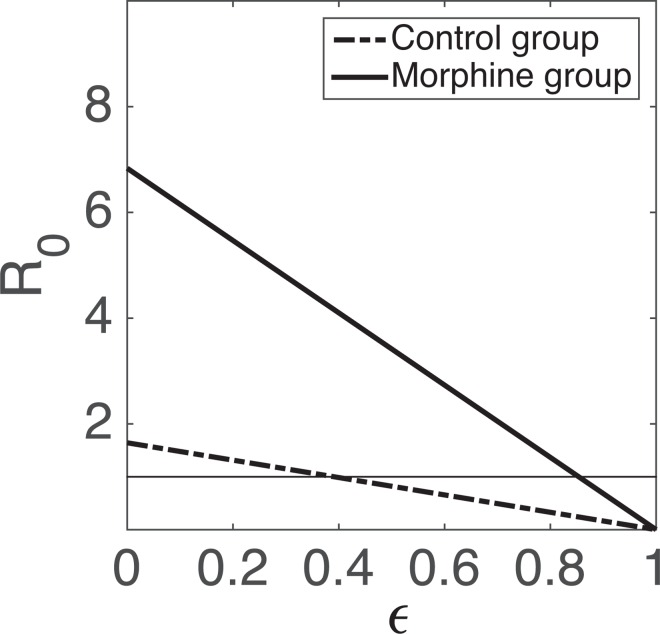
Dependence of the value of *R*_0_ on *ε*, efficacy of ART. Parameters given in Table 2 are used for computation of *R*_0_. The dashed-dot line represents the control group while the solid line represents the morphine group.

While morphine does not seem to play a role in determining whether infection is established or not as *R*_0_ > 1 in both groups, a significantly higher *R*_0_ value in the morphine group comes into play when some interventions, such as antiretroviral therapy as pre-exposure prophylaxis (PrEP), are considered. If *ε* is the efficacy of PrEP, then *ε* > 1 − 1/*R*_0_ is needed for successful control of infection (i.e., for bringing the *R*_0_ value to less than 1). Thus, we estimate that at least 52% effective PrEP can control infection in the control group, while at least 90% efficacy of PrEP is required in the morphine group. Fig 5 shows how *R*_0_ depends on PrEP efficacy. These results suggest that morphine can decrease the effectiveness of PrEP in people who abuse drugs.

**Fig 6:**
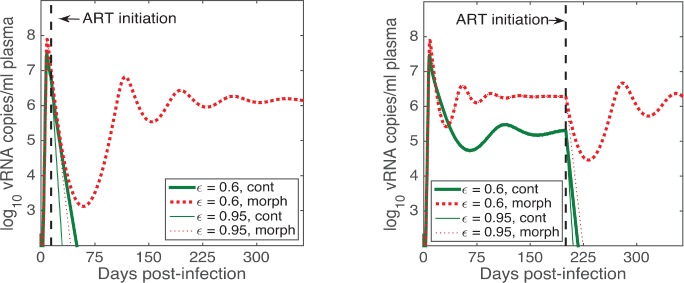
Predicted effects of morphine on antiretroviral therapy. Early ART is initiated 14 days post infection (left) and late ART is initiated 200 days post infection (right). The solid lines represent the control group and the dotted lines represent the morphine group. Two different ART efficacies, 0.6 (medium), and 0.95 (high), are simulated. Parameters given in Table 2 are used for model simulations.

Significant effects of morphine can also be seen in post infection antiretroviral therapy (ART) as demonstrated by model simulations with early ART initiation (14 days post infection) and late ART initiation (200 days post infection) (Fig 6). Whether ART can control viral load or not depends upon the efficacy of ART. While the high ART efficacy, ε = 0.95, can successfully control the virus in both groups, the viral suppression is more effective in the control group than in the morphine group in general (Fig 6). Most importantly, for intermediate level of ART efficacy (for example, 60% in our simulation), ART can suppress the viral load in the control group, but fails to suppress in the morphine group with both early and late ART initiation (Fig 6). Note that the value of *ε* = 0.95 and 0.6 are taken only for demonstration purposes. While current combination ARTs are highly efficacious, it is possible to have lower efficacies due to lack of compliance or emergence of resistance. Further, some combination therapies have been estimated to have efficacies in the 60 to 70% range [63].

### Sensitivity to *V*_0_ and *T*_*h*0_

Due to lack of information about the actual number of virions that initiate infection, we studied how our parameter estimates are affected by the choice of *V*_0_. We considered 500 different values of *V*_0_ selected randomly from 1 to 4 log_10_ viral RNA copies/ml. For each *V*_0_ selected, we estimated parameters for the morphine group as well as the control group. The box and whisker plots of the parameter estimates (S2 Fig) showed that the median change of each estimate remains less than 10% of the base case estimate. Also, based on *in vitro* experiments [41] we chose the initial proportion of *T*_*h*_ cells as 5% (morphine) and 3% (control) of the total target cell. Varying this value from 1 to 15% did not make any significant change in our estimates (less than 5% in each parameter estimate). Thus, our estimates are robust within these ranges of *V*_0_ and *T*_*h*0_.

## Discussion

The dependence of many HIV-infected patients on drugs of abuse such as opiates is an ongoing problem in the post-HAART era. Although injection drug use is one of the important risk factors for HIV infection, with IDU constituting approximately one-third of new AIDS cases in the USA, the relationship between IDU and HIV-1 dynamics remains unclear. In this study, we developed a novel morphine-conditioned viral dynamics model, which is in agreement with experimental data on viral loads and CD4+ T-cell counts in SIV/SHIV infected rhesus macaques (Fig 2 and S1 Fig). This study provides for the first time estimates of SIV viral dynamic parameters in the presence of a drug of abuse. Our model offers insight into how morphine can affect SIV infection and progression.

Our model and parameter estimates support the experimental observation [26, 27, 41, 45, 46] that morphine promotes co-receptor expression in target cells (CD4^+^ T cells), thereby increasing the susceptibility of these cells to HIV/SIV infection. For SIV/SHIV infected rhesus macaques, we estimated the levels of two target cell subpopulations: one group (*T*_*h*_) with an infection rate about 2 orders of magnitude higher than the other group (*T*_*l*_) (p < 0.001). Moreover, our estimates show that the rate of switch between these two subpopulations is greatly affected by morphine (p < 0.005) that causes a three-fold higher rate of transfer from *T*_*l*_ to *T*_*h*_ cells with almost negligible flow of cells in other direction (Table 2). While the precise mechanism by which morphine alters co-receptor expression remains to be determined, the finding of morphine promoting co-receptor expression can be related to the well-established capacity of opioids to alter cytokine release [27, 41, 64]. Morphine may activate the release of cytokines (e.g. tumor necrosis factor-*α* or IL-2) known to stimulate chemokine receptor expression or morphine may inhibit the synthesis of certain chemokines (e.g., RANTES) that cause internalization of co-receptors.

A morphine effect on increasing co-receptor expression is consistent with *in vitro* and *in vivo* experiments [27, 41, 60] as well as the results here on the increased transition from *T*_*l*_ to *T*_*h*_ cells in the morphine-dependent group. Furthermore, based on simulation results in the presence of infection, in the long run, the percentage of *T*_*h*_ target cells in the morphine group can reach an extremely high level (almost 85%, Fig 3). These predicted levels are higher than seen in previous experiments [60] and suggests that either morphine exposure in the absence of SIV infection might not have been long enough to reach the steady state in the experimental studies [27, 41, 60] or the rate of subpopulation switch is different in the presence of SIV infection. If the latter is true, it indicates that not only does morphine alter viral infection, but also viral infection alters the effects of morphine. Further experiments, particularly, on long-term morphine effects in the absence of HIV/SIV infection will be necessary to clarify this issue.

Our estimates show that *R*_0_ is higher in morphine-dependent animals than in control animals, indicating that morphine may induce additional obstacles for intervention strategies such as ART as PrEP. For example, a higher efficacy of ART used for PrEP may be required for morphine-dependent populations than for the general population. We note that the *R*_0_ values estimated for morphine-dependent animals (9.69) and control animals (2.08), although low, both fall within the approximate range of previously estimated *R*_0_ values for HIV/SIV [29, 65]. Therefore, we cannot be definite on whether the elevated *R*_0_ value in the morphine group is due to the presence of morphine or some other causes such as variation among hosts. Further analysis with larger data sets is needed to better understand the effects of morphine on *R*_0_.

Using steady state analysis and model simulations, we studied the long-term dynamics. Since *R*_0_ > 1 in both the morphine and control groups, the infection free steady state is unstable and infection was established in these animals as reported earlier [16]. A marked difference was seen in the steady state viral load levels in these two groups; with the viral load steady state being 1.0 log_10_ higher in the morphine group than in the control group (Fig 3). The dynamics predicted by the model also shows that the total CD4+ T-cell count decreases faster in the morphine group than in the control group, particularly during the acute phase of infection. The model predicts that there is a higher CD4+ T-cell count drop in the morphine group than in the control group (90% drop in morphine group vs. 82% drop in control group after a year of SIV infection, Fig 4). This drop is not statistically significant when all animals are included in the analysis, but it becomes significant when animal M4 with an unusually high CD4+ T-cell count is excluded from the analysis. While we need more data to accurately evaluate the significance of the predicted CD4+ T-cell count drop, the current results suggest that the morphine may exacerbate the progression and pathogenesis of HIV/SIV infection in agreement with the experimental observations.

Effect of morphine is also clearly revealed in our model simulations in the presence of ART (Fig 6); morphine conditioning makes it harder to control the viral load with both early and late ART initiations. Most notably, for an intermediate level of drug efficacy, ART may fail to control the viral load in morphine-dependent individuals but control the viral load in non-dependent individuals. This suggests that to achieve treatment success, the ART protocol for patients with a drug addiction may have to be different than for patients without drug addictions.

We acknowledge several limitations of this study. Our parameter estimates are based on a limited data set. A larger data set obtained in both the presence and absence of HIV/SIV infection would help us gain more confidence in the estimated parameter values and related results. Our model also does not distinguish between the two main HIV/SIV co-receptors, CCR5 and CXCR4. While morphine increases cell surface levels of both of these co-receptors [41], the level of expression induced by morphine may be different between the two; one study [60] indicates that the effect of morphine on CCR5 expression is more pronounced compared to CXCR4 expression. Since 2 of the 3 viruses used in this experiment are either dual-tropic or R5-tropic, our results may be more applicable to CCR5 viruses. Our results may need to be considered carefully in the case of X4 viruses if CXCR4 expression responds differently to morphine exposure.

Our model is focused on the effect of morphine on inducing co-receptor expression. Other physiological alterations induced by morphine could also have an impact on viral kinetics. For example, morphine could lead to differences in inflammation status, or in the viral dynamics across the blood-brain barrier, or in immune responses. Here we did not try to study exhaustively all these other factors. We did however analyze the potential for immune effects to explain the differences between the observed viral dynamics in the presence and absence of morphine (see S2 Text). We fitted two immune response models, including cytolytic or non-cytolytic effects, but found that these provided much poorer fits to the data than our model based on differences in target cell susceptibility. Indeed, using AICc, we showed that the data provide much better support for the latter model. While these basic models (as well as the experimental results discussed above) do not support noticeable effects of morphine on immune responses, a more detailed analysis using models including immune responses [34–37] and tissue compartments can be developed, but would involve additional parameters and require more data to validate. In this context, it is interesting that we found a statistically significant lower *δ*, the death rate of productively infected cells, in the morphine group (p<0.05). This seems to indicate that a less vigorous immune response may also be a result of morphine, although it is not sufficient to explain the difference in viral dynamics based on our analyses of the immune response models. Thus, quantifying other effects of morphine on the immune system and on viral dynamics via modeling will continue to be an active field of investigation. For example, we suggest that an experiment in which ART is given to morphine-conditioned vs. control animals may provide a better indication for differences in the death rate of productively infected cells.

## Supporting Information

S1 Text Mathematical analysis of the model. (PDF)

S2 Text Analyses of immune response models with cytolytic or non-cytolytic effects. (PDF)

S1 Table 95% Confidence intervals for the estimated model parameters. (PDF)

S1 Fig Model fit to the data from individual monkeys. (PDF)

S2 Fig Sensitivity of the fitted model parameters to the initial viral load *V*_0_. (PDF)
