# Supplementary material for: Modeling the Effects of Morphine on Simian Immunodeficiency Virus Dynamics
Source: PLoS Comput Biol. 2016 Sep 26;12(9):e1005127. doi: 10.1371/journal.pcbi.1005127 (PMC5036892; doi:10.1371/journal.pcbi.1005127)
Supplement: S1 Text — (PDF) [file pcbi.1005127.s001.pdf]

# Text S1. Mathematical Analysis of the model

## Change in $T_0$ affects only parameters $\lambda$ and $p$ of the model system

We scale the variables $T_l$, $T_h$, and $I$ in the model system as $\hat{T}_l = T_l/T_0$, $\hat{T}_h = T_h/T_0$, and $\hat{I} = I/T_0$. Then the model system reduces to

$$\frac{d\hat{T}_l}{dt} = \frac{\lambda}{T_0} + q\hat{T}_h - d\hat{T}_l - r\hat{T}_l - \beta_l V\hat{T}_l, \qquad \hat{T}_l(0) = \frac{T_{l0}}{T_0},$$

$$\frac{d\hat{T}_h}{dt} = r\hat{T}_l - d\hat{T}_h - \beta_h V\hat{T}_h - q\hat{T}_h, \qquad \hat{T}_h(0) = \frac{T_{h0}}{T_0},$$

$$\frac{d\hat{I}}{dt} = \beta_l V\hat{T}_l + \beta_h V\hat{T}_h - \delta\hat{I}, \qquad \hat{I}(0) = \frac{I_0}{T_0},$$

$$\frac{dV}{dt} = pT_0 I - cV, \qquad V(0) = V_0.$$

Now, applying transformations $\lambda \to \lambda T_0$ and $p \to p/T_0$, we obtain the original system. Hence, change in $T_0$ affects only parameters $\lambda$ and $p$ of the model system.

## Calculation of the basic reproduction number using the next generation matrix

In this section, we derive $R_0$, the reproduction number, using the next generation matrix method [1-3]. It is easy to see that the model system has exactly one infection-free equilibrium $E^0 = (T_l^0, T_h^0, I^0, V^0) = \left(\frac{\lambda(d+q)}{d(d+r+q)}, \frac{\lambda r}{d(d+r+q)}, 0, 0\right)$, and the equations for the infected cell and virus compartments of the linearized system of the model at $E^0$ take the form:

$$\frac{dI}{dt} = -\delta I + \frac{\lambda}{d(d+r+q)}[\beta_l(d+q) + \beta_h r]V, \qquad (S1)$$

$$\frac{dV}{dt} = pI - cV. \qquad (S2)$$

We now introduce the matrices:

$$F = \begin{pmatrix} 0 & \frac{\lambda}{d(d+r+q)}[\beta_l(d+q) + \beta_h r] \\ 0 & 0 \end{pmatrix},$$

and

$$\mathcal{V} = \begin{pmatrix} \delta & 0 \\ -p & c \end{pmatrix}.$$

These expressions give

$$FV^{-1} = \begin{pmatrix} \frac{\lambda p}{\delta cd(d+r+q)}[\beta_l(d+q)+\beta_h r] & \frac{\lambda}{cd(d+r+q)}[\beta_l(d+q)+\beta_h r] \\ 0 & 0 \end{pmatrix}.$$

Then $R_0$ corresponds to the spectral radius of $FV^{-1}$:

$$R_0 = \rho(FV^{-1}) = \frac{\lambda p}{\delta cd(d+r+q)}[\beta_l(d+q)+\beta_h r].$$

**Proof of "$E^0$ is locally asymptotically stable if $R_0 < 1$ and unstable if $R_0 > 1$"**

Jacobian of the model system linearized around $E^0$ is:

$$J^0 = \begin{pmatrix} X_{2\times 2} & Y_{2\times 2} \\ 0_{2\times 2} & Z_{2\times 2} \end{pmatrix},$$

where

$$X = \begin{pmatrix} -d-r & q \\ r & -d-q \end{pmatrix},$$

$$Y = \begin{pmatrix} 0 & \frac{-\beta_l \lambda (d+q)}{d(d+r+q)} \\ 0 & \frac{-\beta_h \lambda r}{d(d+r+q)} \end{pmatrix},$$

$$Z = \begin{pmatrix} -\delta & \frac{\lambda}{d(d+r+q)}[\beta_l(d+q)+\beta_h r] \\ p & -c \end{pmatrix}.$$

Here, eigenvalues of $J^0$ are given by eigenvalues of the matrices $X$ and $Z$.

Since $tr(A) = -(2d+r+q) < 0$ and $\det(A) = d(d+r+q) > 0$, both eigenvalues of $X$ are negative.
Also,

$$tr(Z) = -(\delta + c) < 0.$$
$$\det(Z) = \delta c[1 - R_0] > 0 \quad \text{iff} \quad R_0 < 1.$$

This implies that eigenvalues of $Z$, and so eigenvalues of $J^0$, are negative if and only if $R_0 < 1$. Hence, $E^0$ is locally asymptotically stable if $R_0 < 1$ and unstable if $R_0 > 1$

**Proof of "$E^*$ exists if $R_0 > 1$, and $E^*$, if it exists, is locally asymptotically stable"**

For the analysis of the infected steady state during morphine conditioning, we assume $\beta_l \ll \beta_h$ and $q \ll r$ as given by our parameter estimates to simplify the model. In this case, $E^*$ is given by

$$E^* = (T_l^*, T_h^*, I^*, V^*) = \left(\frac{\lambda}{d+r}, \frac{\delta c}{\beta_h p}, \frac{dc}{\beta_h p}(R_0 - 1), \frac{d}{\beta_h}(R_0 - 1)\right).$$

Clearly, $E^*$ exists if $R_0 > 1$. Jacobian of the model system linearized around $E^*$ is:

$$J^* = \begin{pmatrix} -d-r & 0_{1\times 3} \\ A_{3\times 1} & B_{3\times 3} \end{pmatrix},$$

where

$$A = \begin{pmatrix} r \\ 0 \\ 0 \end{pmatrix}, \qquad B = \begin{pmatrix} -dR_0 & 0 & -\delta c/p \\ d(R_0 - 1) & -\delta & \delta c/p \\ 0 & p & -c \end{pmatrix}.$$

One eigenvalue of $J^*$ is $-(d+r) < 0$, and other three eigenvalues are given by eigenvalues of the matrix $B$. The eigenvalue, $\xi$, of the matrx $B$ is obtained by solving

$$\det(B - \xi I_{3\times 3}) = 0.$$

$$\text{i.e., } \begin{vmatrix} -dR_0 - \xi & 0 & -\delta c/p \\ d(R_0 - 1) & -\delta - \xi & \delta c/p \\ 0 & p & -c - \xi \end{vmatrix} = 0.$$

This implies

$$a_3\xi^3 + a_2\xi^2 + a_1\xi + a_0 = 0,$$

where $a_0 = \delta c d(R_0 - 1)$, $a_1 = dR_0(\delta + c)$, $a_2 = dR_0 + \delta + c$, $a_3 = 1$. If $R_0 > 1$, each $a_i > 0$, $i = 0,1,2,3$. Also, it is easy to show that $a_2 a_1 - a_3 a_0 > 0$. Therefore, by Routh-Hurwitz criterion, all eigenvalues of $B$, and so all eigenvalues of $J^*$, are negative. Hence, if $R_0 > 1$, $E^*$ exists and is locally asymptotically stable.

## References


1. Diekmann O, Heesterbeek JA, Roberts MG (2009) The construction of next-generation matrices for compartmental epidemic models. J R Soc Interface 7: 873–885.

2. van den Driessche P, Watmough J (2002) Reproduction numbers and sub-threshold endemic equilibria for compartmental models of disease transmission. Math Biosci 180: 29–48.

3. Heffernan JM, Smith RJ, Wahl LM (2005) Perspectives on the basic reproductive ratio. J R Soc Interface 2: 281–293.
