# Supplementary material for: Modeling the Effects of Morphine on Simian Immunodeficiency Virus Dynamics
Source: PLoS Comput Biol. 2016 Sep 26;12(9):e1005127. doi: 10.1371/journal.pcbi.1005127 (PMC5036892; doi:10.1371/journal.pcbi.1005127)
Supplement: S2 Text — (PDF) [file pcbi.1005127.s002.pdf]

Text S2. Analyses of immune response models with cytolytic or non-cytolytic effects

We consider the basic viral dynamics model with uninfected target cells, T, infected target cells, I, and free virus particles, V. We then introduce a population of effector cells, E, that can act via cytolytic or non-cytolytic mechanisms. We model the cytolytic effect by increasing infected cell killing at a rate proportional to the size of the effector cell population, i.e. we replace $\delta \to \delta + \delta_E E$. Thus the cytolytic immune response model (CIR model) is

$$\frac{dT}{dt} = \lambda - dT - \beta VT, \qquad T(0) = T_0,$$
$$\frac{dI}{dt} = \beta VT - (\delta + \delta_E E)I, \qquad I(0) = I_0,$$
$$\frac{dV}{dt} = pI - cV, \qquad V(0) = V_0,$$
$$\frac{dE}{dt} = \frac{\xi I}{K+I}E - d_E E, \qquad E(0) = E_0.$$

Here, δ_E is the rate of infected cell killing by effector cells. The effector cells are stimulated proportionally to I and E, with a rate constant ξ and with a saturation function dependent on the level of infected cells with a half-maximal stimulation threshold K. d_E represents the loss rate of effector cells.

Similarly, we model the non-cytolytic effect by decreasing the rate of viral production by infected cells, i.e. we use $p \to p/(1 + p_E E)$, where p_E is a scaling factor. Thus the non-cytolytic immune response model (NIR model) is

$$\frac{dT}{dt} = \lambda - dT - \beta VT, \qquad T(0) = T_0,$$
$$\frac{dI}{dt} = \beta VT - \delta I, \qquad I(0) = I_0,$$
$$\frac{dV}{dt} = \frac{p}{1 + p_E E}I - cV, \qquad V(0) = V_0,$$
$$\frac{dE}{dt} = \frac{\xi I}{K+I}E - d_E E, \qquad E(0) = E_0.$$

We fitted the CIR model, NIR model, and TCS model (target cell population switch model) to the viral load and CD4 data from the morphine and control groups. For model comparison we computed the small sample size corrected Akaike's information criterion (AICc) using the formula

$$AIC_C = N \ln \frac{J}{N} + \frac{2N(N_p + 1)}{N - N_p - 2}$$

where J, N and N_p are, respectively, the sum of the squared residuals, the number of data points and the number of parameters estimated in each case. For the CIR model, the fitted parameters are $\lambda, \beta, \delta, \delta_E, \xi, K$ and d_E, while for the NIR model, the fitted parameters are $\lambda, \beta, \delta, p_E, \xi, K$ and

d_E. We also tested a model with $K = 0$, which did not provide good fits. As we describe in detail in the main text, the fitted parameters in the TCS model are λ, β_l, β_h, δ, *r*, *q*. The other parameters (*d*, *c*, and *p*) were fixed at the same values in all models (see main text), and in the immune models E_0=500 ml^{-1}. The best fits of the models are shown in Fig. 1_S2_Text, AIC_C values are given in Table 1_S2_Text, and the best fit parameters for the immune models are shown in Table 2_S2_Text and Table 3_S2_Text. The AIC_c values show that the TCS model is preferred over the CIR and NIR models. Moreover, as seen in the figure below, the structure of the immune response models leads to much poorer fits.

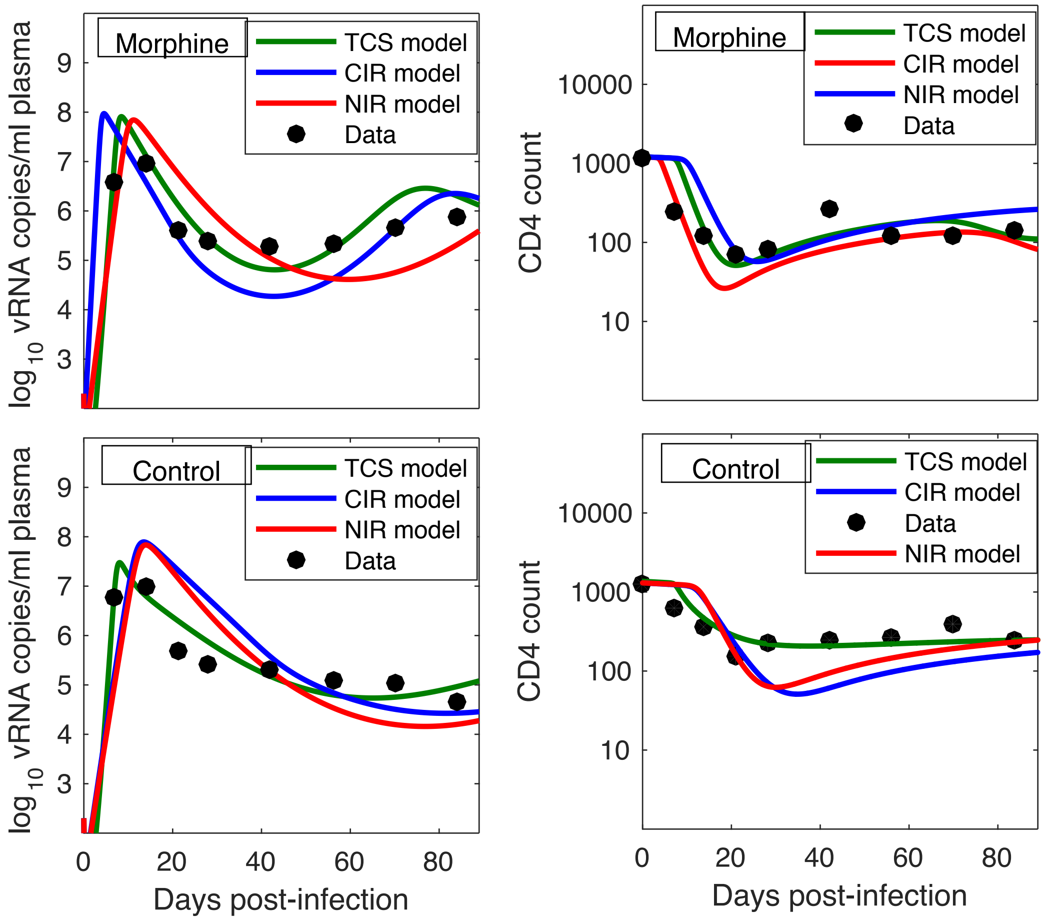

Figure 1_S2_Text. *Best-fit viral load (left column) and CD4 count (right column) dynamics predicted by the models (lines) along with the mean* log_{10} *viral load and CD4+ T-cell count data (circles) for the morphine group (first row) and the control group (second row).*

We also plot the dynamics of $\delta_E E$ predicted by the CIR model (Fig. 2_S2_Text). In both the morphine and control groups, $\delta_E E$ is much smaller than the value of δ. Similarly, predictions of the NIR model show that the magnitude of $p_E E$ is much smaller than 1 (Fig. 2_S2_Text).

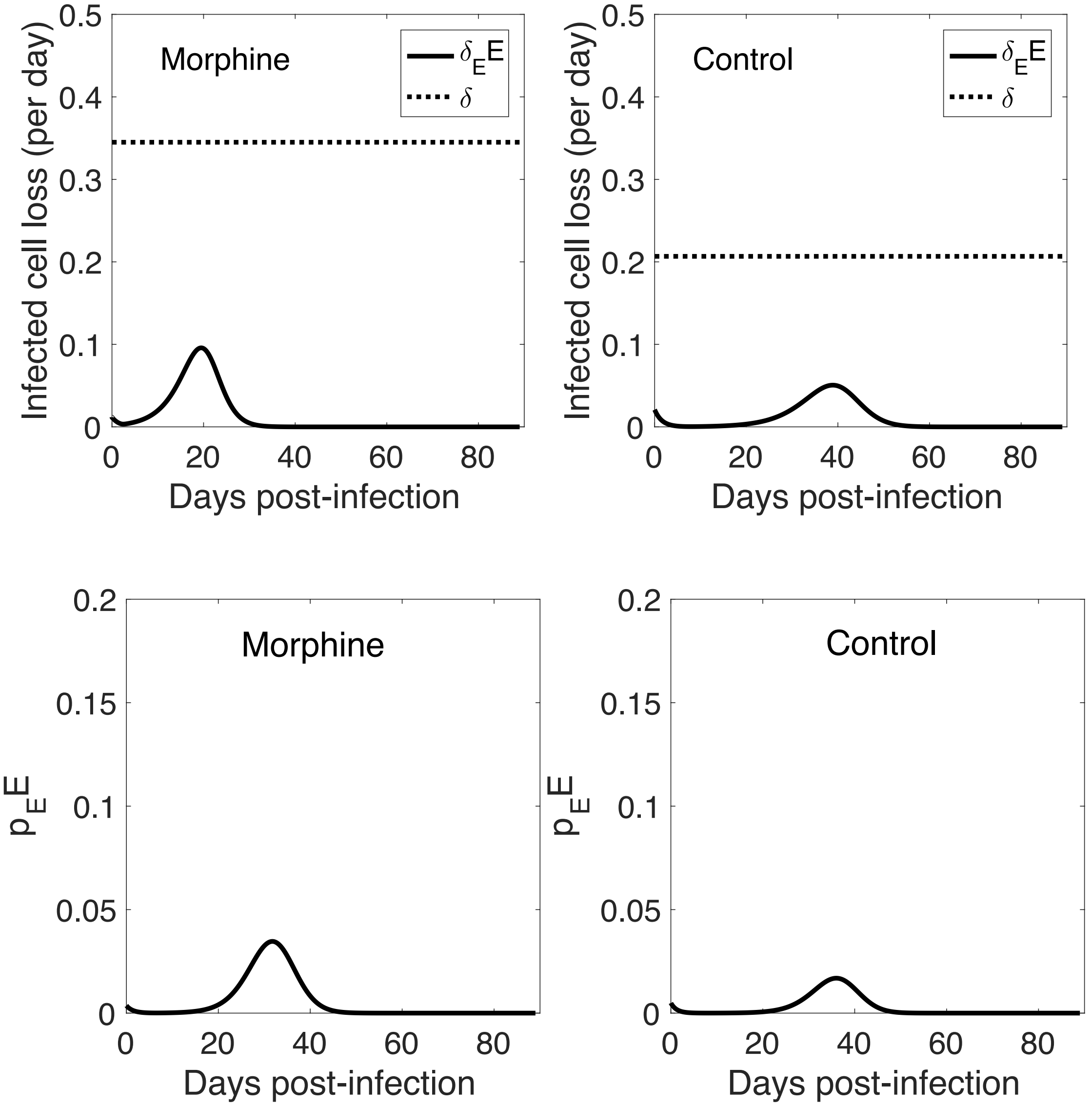

Figure 2_S2_Text. First row (CIR model): *Dynamics of the per capita infected cell killing due to effector cell, $\delta_E E$, and the per capita infected cell death, δ. Second row (NIR model): Dynamics of reduction in viral production, $p_E E$.*

In addition to fitting the two groups separately, we also fitted the CIR and NIR models to both morphine and control groups together. In this case, we allowed only the immune response related parameters to be different between the groups, keeping the other parameters the same in both groups. We have a larger data set with the two groups combined allowing us to fit a larger number of parameters. The parameters fitted were $\lambda, \beta, \delta, {\delta_E}^M, \xi^M, K^M, {d_E}^M, {\delta_E}^C, \xi^C, K^C, {d_E}^C$ for the CIR model and $\lambda, \beta, \delta, {p_E}^M, \xi^M, K^M, {d_E}^M, {p_E}^C, \xi^C, K^C, {d_E}^C$ for the NIR model. Superscripts M and C stand for morphine and control, respectively. The best fit of the models to the viral load and CD4 data for both groups are shown in Fig. 3_S3_Text, and the AIC_C and the sum of squared residual (SSR) values are given in Table 1_S2_Text. Neither the AIC_C nor SSR value was

improved with this approach, further supporting that the immune response is not significantly different between the two groups of monkeys.

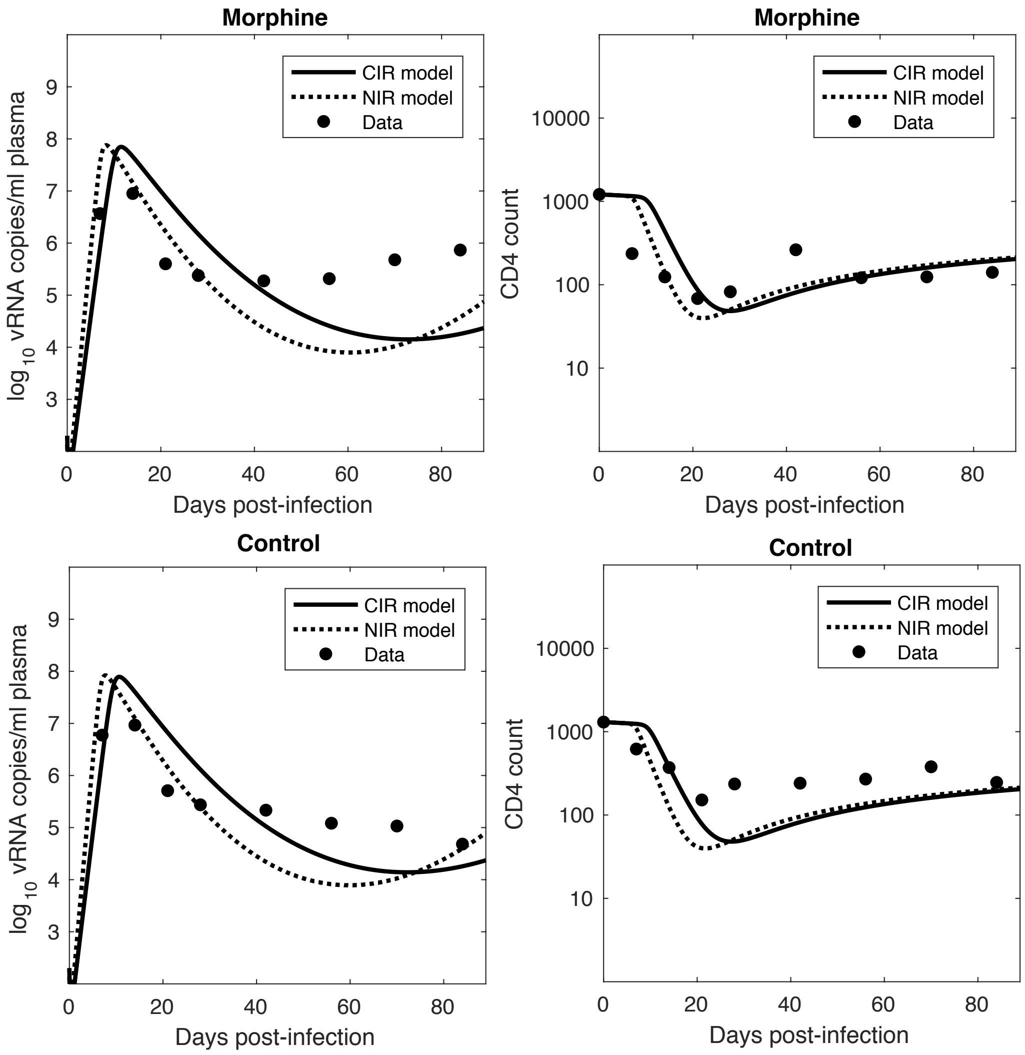

Figure 3_S2_Text. *Fitting of CIR and NIR models to morphine and control groups data together. Best-fit viral load (left column) and CD4 count (right column) dynamics predicted by the models (lines) along with the mean log_{10} viral load and CD4 count data (filled small circles) for the morphine group (first row) and the control group (second row).*

Table 1_S2_Text:
Akaike's information criteria (AIC_C) and sum of squared residuals (SSR) for model fits to the data.

Model	Morphine Group		Control Group		Both Groups Together	
	SSR	AIC_C	SSR	AIC_C	SSR	AIC_C
CIR model	4.12	14.97	9.24	27.80	15.12	16.42
NIR model	5.32	18.95	8.99	27.36	15.45	17.12
TCS model	2.02	-5.07	1.04	-15.59	N/A	N/A

Table 2_S2_Text: Estimated parameters for CIR model.

Parameters	Morphine	Control
λ	3280	3700
β	3.83×10^{-8}	1.21×10^{-8}
δ	0.37	0.20
δ_E	2.34×10^{-5}	4.17×10^{-5}
ξ	0.86	0.83
K	100	99
d_E	0.61	0.63

Table 3_S2_Text: Estimated parameters for NIR model.

Parameters	Morphine	Control
λ	5440	5080
β	1.57×10^{-8}	1.20×10^{-8}
δ	0.30	0.28
p_E	7.22×10^{-6}	9.78×10^{-6}
ξ	1.03	1.11
K	106	95
d_E	0.71	0.80
