# Supplementary material for: Modeling the Effects of Morphine on Simian Immunodeficiency Virus Dynamics
Source: PLoS Comput Biol. 2016 Sep 26;12(9):e1005127. doi: 10.1371/journal.pcbi.1005127 (PMC5036892; doi:10.1371/journal.pcbi.1005127)
Supplement: S1 Table — (PDF) [file pcbi.1005127.s003.pdf]

Table S1. 95% Confidence intervals for the estimated model parameters. 95% Confidence Intervals Obtained by a Bootstrap Procedure (Morphine group: M1-M6; Control group: C1-C6). Last row for each group represents estimates for geometric mean viral load and mean CD4 count.

**Monkey**		$\boldsymbol{\delta}$	$\boldsymbol{\lambda}$	$\boldsymbol{r}$	$\boldsymbol{q}$	$\boldsymbol{\beta_l}$	$\boldsymbol{\beta_h}$
M1	Lower	0.33	1.48×10^{3}	0.61	2.69×10^{-7}	5.62×10^{-9}	5.72×10^{-8}
	Upper	0.45	2.10×10^{3}	0.77	4.99×10^{-7}	7.69×10^{-9}	9.52×10^{-7}
M2	Lower	0.30	2.06×10^{3}	0.55	3.73×10^{-7}	3.62×10^{-10}	7.20×10^{-8}
	Upper	0.39	2.76×10^{3}	0.70	5.94×10^{-7}	6.03×10^{-10}	1.20×10^{-7}
M3	Lower	0.38	7.06×10^{3}	0.38	3.63×10^{-6}	5.70×10^{-9}	6.60×10^{-8}
	Upper	0.48	9.43×10^{3}	0.46	5.92×10^{-6}	7.28×10^{-9}	9.85×10^{-8}
M4	Lower	0.51	7.06×10^{3}	0.52	2.39×10^{-7}	5.24×10^{-9}	1.27×10^{-8}
	Upper	0.65	9.71×10^{3}	0.79	2.41×10^{-7}	1.08×10^{-8}	1.90×10^{-8}
M5	Lower	0.48	2.55×10^{3}	0.29	3.43×10^{-8}	1.14×10^{-11}	8.16×10^{-8}
	Upper	0.66	3.42×10^{3}	0.38	4.53×10^{-8}	1.90×10^{-11}	1.22×10^{-7}
M6	Lower	0.30	1.10×10^{3}	0.50	3.53×10^{-8}	5.08×10^{-10}	7.57×10^{-8}
	Upper	0.39	1.48×10^{3}	0.66	4.63×10^{-8}	6.53×10^{-10}	1.23×10^{-7}
Mean Data	Lower	0.34	5.17×10^{3}	0.48	4.30×10^{-7}	5.13×10^{-10}	2.78×10^{-8}
	Upper	0.41	5.18×10^{3}	0.52	1.23×10^{-6}	5.56×10^{-10}	3.03×10^{-8}
C1	Lower	0.58	2.96×10^{3}	0.13	0.16	8.15×10^{-12}	5.81×10^{-8}
	Upper	0.80	3.69×10^{3}	0.16	0.22	1.30×10^{-11}	7.36×10^{-8}
C2	Lower	0.57	3.22×10^{3}	0.18	0.16	7.40×10^{-10}	5.62×10^{-8}
	Upper	0.80	3.65×10^{3}	0.21	0.22	9.26×10^{-10}	7.18×10^{-8}
C3	Lower	0.35	1.84×10^{3}	0.17	0.21	6.22×10^{-10}	7.41×10^{-8}
	Upper	0.43	2.04×10^{3}	0.23	0.28	7.82×10^{-10}	9.76×10^{-8}
C4	Lower	0.61	3.20×10^{3}	0.13	0.19	3.82×10^{-9}	1.24×10^{-7}
	Upper	0.84	4.28×10^{3}	0.16	0.27	6.37×10^{-9}	2.07×10^{-7}
C5	Lower	0.58	3.39×10^{3}	0.15	0.19	3.66×10^{-11}	4.87×10^{-8}
	Upper	0.82	4.34×10^{3}	0.19	0.26	5.18×10^{-11}	6.53×10^{-8}
C6	Lower	0.58	2.83×10^{3}	0.13	0.17	7.57×10^{-11}	6.47×10^{-8}
	Upper	0.87	3.48×10^{3}	0.17	0.25	1.08×10^{-10}	8.78×10^{-8}
Mean Data	Lower	0.72	3.68×10^{3}	0.14	0.22	9.8×10^{-11}	7.73×10^{-8}
	Upper	0.79	3.69×10^{3}	0.16	0.25	1.19×10^{-10}	8.45×10^{-8}
