# Supplementary material for: Modeling the Effects of Morphine on Simian Immunodeficiency Virus Dynamics
Source: PLoS Comput Biol. 2016 Sep 26;12(9):e1005127. doi: 10.1371/journal.pcbi.1005127 (PMC5036892; doi:10.1371/journal.pcbi.1005127)
Supplement: S1 Fig — (PDF) [file pcbi.1005127.s004.pdf]

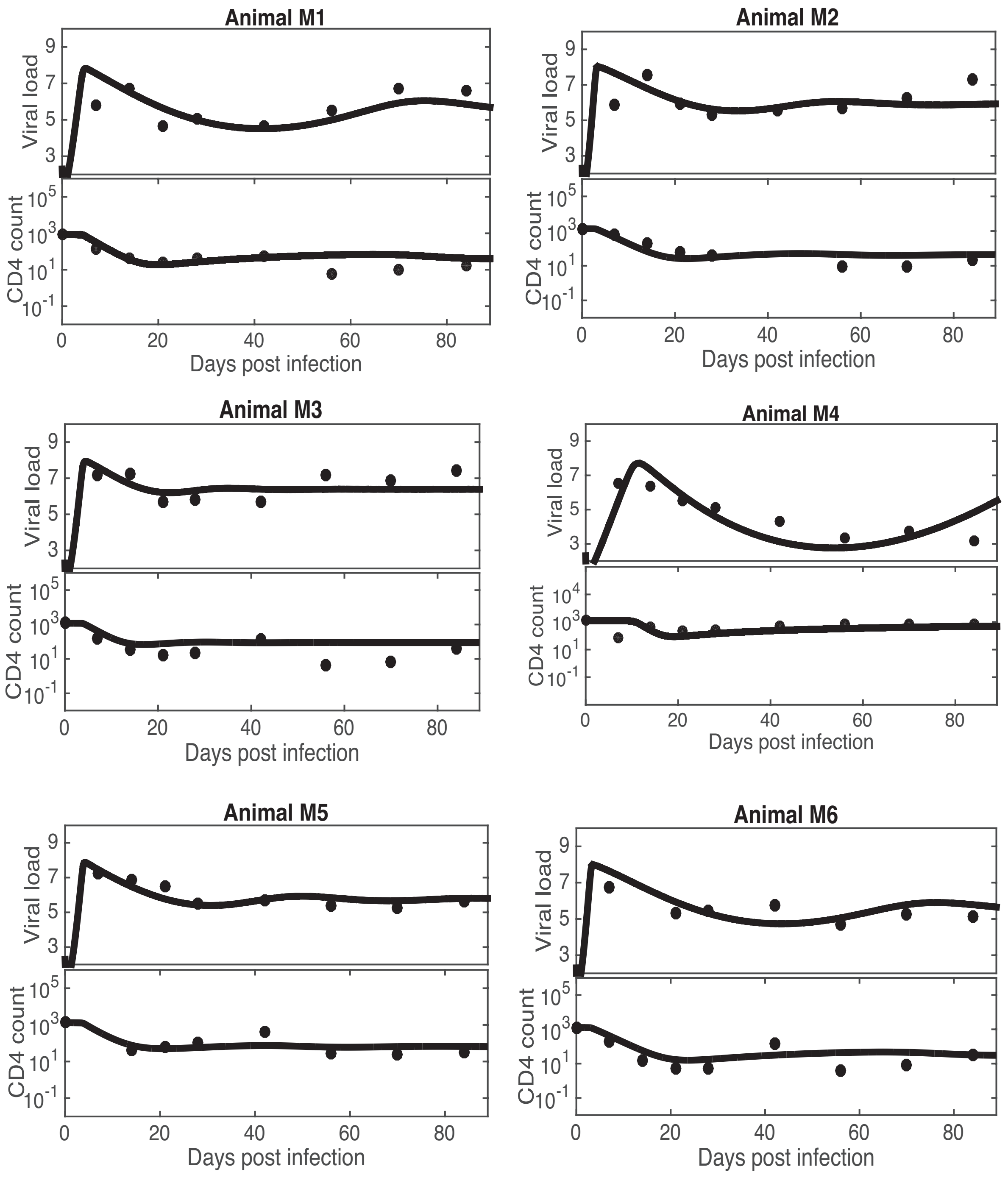

Figure S1a. Model fit to the data from individual monkeys in the morphine-group. In each monkey, the upper graph represents viral load and the lower graph represents CD4 count. The solid lines indicate the model prediction with best-estimated parameters and filled small circles indicate the experimental data.

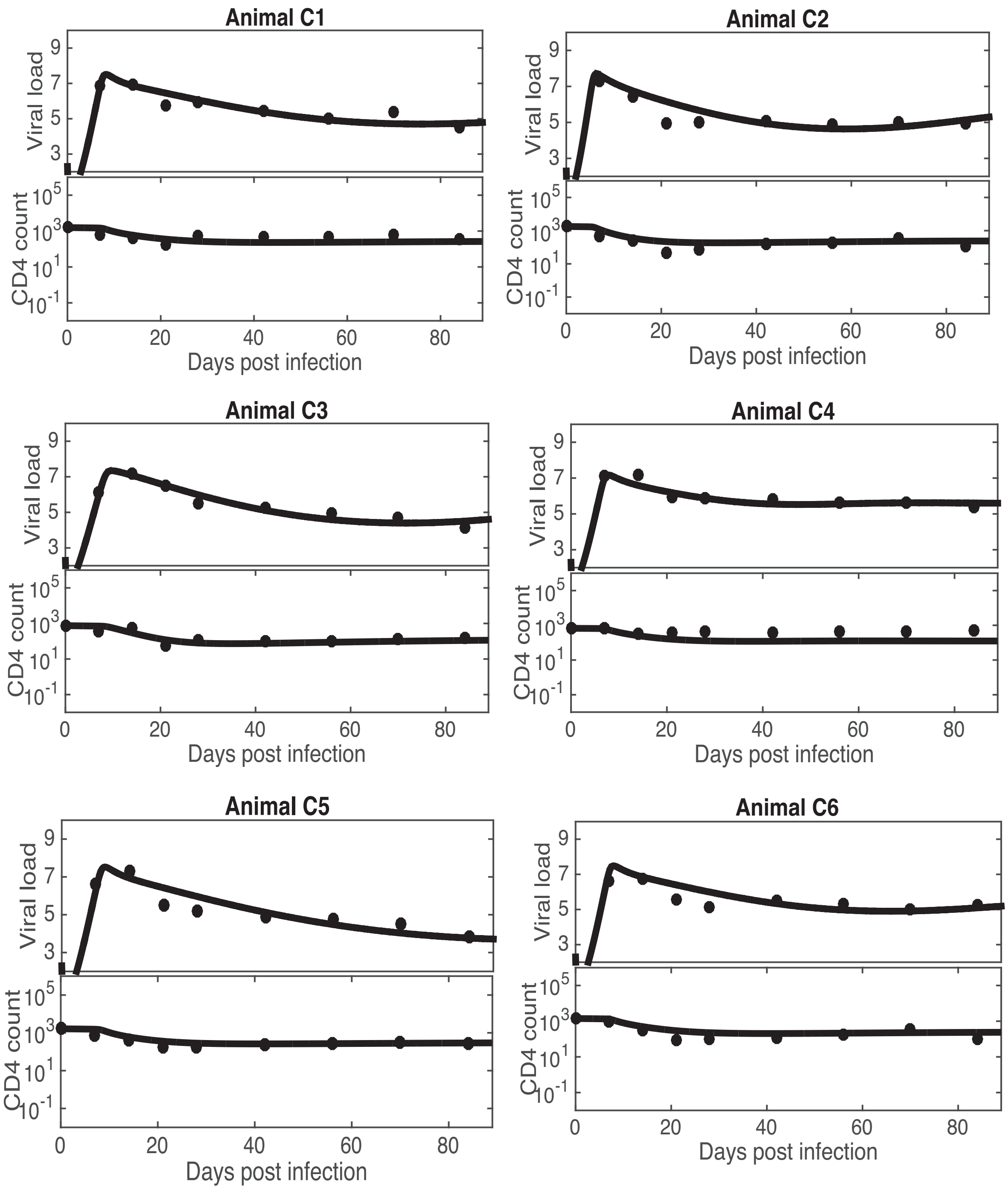

Figure S1b. Model fit to the data from individual monkeys in the control-group. In each monkey, the upper graph represents viral load and the lower graph represents CD4 count. The solid lines indicate the model prediction with best-estimated parameters and filled small circles indicate the experimental data.
