# Supplementary material for: Modeling the Effects of Morphine on Simian Immunodeficiency Virus Dynamics
Source: PLoS Comput Biol. 2016 Sep 26;12(9):e1005127. doi: 10.1371/journal.pcbi.1005127 (PMC5036892; doi:10.1371/journal.pcbi.1005127)
Supplement: S2 Fig — (PDF) [file pcbi.1005127.s005.pdf]

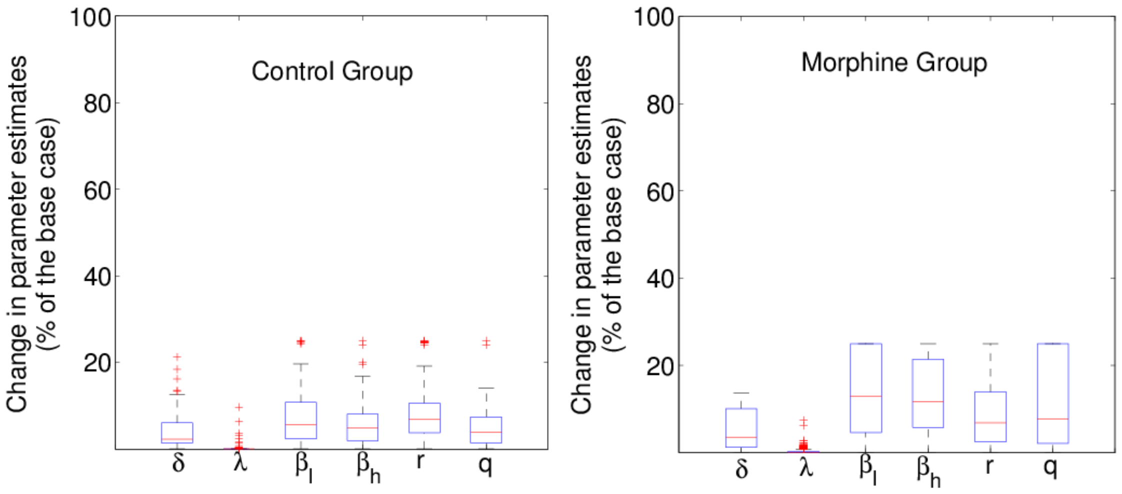

Figure S2. Sensitivity of the fitted model parameters to the initial viral load V_0. Box and Whisker plots showing changes in parameter estimates for 500 different values of V_0 selected randomly between 1 $\log_{10}$ and 4 $\log_{10}$.
